# Quantitative hypermorphic *FAM111A* alleles cause autosomal recessive Kenny-Caffey syndrome type 2 and osteocraniostenosis

**DOI:** 10.1172/jci.insight.186862

**Published:** 2025-02-11

**Authors:** Dong Li, Niels Mailand, Emma Ewing, Saskia Hoffmann, Richard C. Caswell, Lewis Pang, Jacqueline Eason, Ying Dou, Kathleen E. Sullivan, Hakon Hakonarson, Michael A. Levine

**Affiliations:** 1 Center for Applied Genomics, The Children’s Hospital of Philadelphia, Philadelphia, Pennsylvania, USA.; 2 Department of Pediatrics, University of Pennsylvania Perelman School of Medicine, Philadelphia, Pennsylvania, USA.; 3 The Novo Nordisk Foundation Center for Protein Research, University of Copenhagen, Denmark.; 4 Exeter Genomics Laboratory, Royal Devon University Healthcare NHS Foundation Trust, Exeter, United Kingdom.; 5 Department of Clinical Genetics, Nottingham University Hospitals NHS Trust, Nottingham, United Kingdom.; 6 Division of Allergy and Immunology and; 7 Division of Endocrinology and Diabetes and Center for Bone Health, The Children’s Hospital of Philadelphia, Philadelphia, Pennsylvania, USA.

**Keywords:** Endocrinology, Genetics, Genetic diseases

## Abstract

Kenny-Caffey syndrome (KCS) is a rare genetic disorder characterized by extreme short stature, cortical thickening and medullary stenosis of tubular bones, facial dysmorphism, abnormal T cell function, and hypoparathyroidism. Biallelic loss-of-function variants in *TBCE* cause autosomal recessive type 1 KCS (KCS1). By contrast, heterozygous missense variants in a restricted region of the *FAM111A* gene have been identified in autosomal dominant type 2 KCS (KCS2) and a more severe lethal phenotype, osteocraniostenosis (OCS); these variants have recently been shown to confer a gain of function. In this study, we describe 2 unrelated children with KCS and OCS who were homozygous for different *FAM111A* variant alleles that result in replacement of the same residue, Tyr414 (c.1241A>G, p.Y414C and c.1240T>A, p.Y414N), in the mature FAM111A protein. Their heterozygous relatives are asymptomatic. Functional studies of recombinant FAM111A^Y414C^ demonstrated normal dimerization and a mild gain-of-function effect. This study provides evidence that both biallelic and monoallelic variants of *FAM111A* with varying degrees of activation can lead to dominant or recessive KCS2 and OCS.

## Abstract 2

Two recessive *FAM111A* variants in KCS2 and OCS

## Introduction

Kenny-Caffey syndrome (KCS) is an uncommon disorder that is characterized by skeletal defects that include severe proportionate short stature, gracile bones with thickened cortices and medullary stenosis, and cranial defects such as delayed fontanelle closure, ocular abnormalities, and facial dysmorphism. Many patients also have endocrine defects, most commonly hypoparathyroidism but also rarely various pituitary abnormalities (1, 2) and recurrent bacterial infections that have been ascribed to a putative T cell immune defect (3, 4).

The original descriptions of KCS identified an autosomal dominant pattern of inheritance (1, 5), which appears to be the most common form of the disorder that is now termed KCS2 (MIM 127000). Later reports described a second form of KCS with apparent autosomal recessive transmission that is termed KCS1 (MIM 244460); this form differs from KCS2 by the presence of prenatal growth restriction and mild to moderate intellectual disability (4, 6–11). KCS1 overlaps with the autosomal recessive Sanjad-Sakati syndrome (also known as hypoparathyroidism-growth retardation-dysmorphism syndrome [HRD], OMIM 241410), a disorder that has been reported principally in people of Arab descent living in the Middle East and in which medullary stenosis is far less common than KCS1 (10, 12–14).

The identification of recessive variants in the *TBCE* gene at chromosome 1q42.3 as the molecular defect in KCS1 and HRD showed that these 2 overlapping syndromes were allelic disorders in which identical molecular defects could lead to different clinical phenotypes (3). Nearly all affected participants of Arab descent in the Middle East have been found to be homozygous for a 12 bp deletion in the second coding exon of the *TBCE* gene, regardless of whether they have KCS1 or HRD and share an ancestral haplotype that has suggested a common founder variant (10). Nevertheless, at present, there is no explanation for the phenotypic discrepancy between KCS1 and HRD (3). *TBCE* encodes tubulin-specific chaperone E, a protein that is required for α- and β-tubulin dimerization and microtubule polymerization (3, 13). In addition to HRD and KCS1, hypomorphic *TBCE* alleles have also been identified in patients with autosomal recessive Progressive Encephalopathy with Amyotrophy and Optic Atrophy (PEAMO; OMIM 617207), a distinctive neurodevelopmental/neurodegenerative disorder (15).

Heterozygous missense variants of the *FAM111A* gene (16–21) on chromosome 11q12.1 are now recognized as the molecular basis for KCS2. Moreover, similar missense variants in *FAM111A* have also been identified in patients with osteocraniostenosis (OCS) — also termed gracile bone dysplasia (GCLEB; OMIM 602361), a severe skeletal condition that is often fatal (16, 20) — and in a patient with severe growth impairment thought to have primary deficiency of insulin-like growth factor 1 (22). *FAM111A* encodes a putative serine protease (Family with sequence similarity 111 member A) that protects replication forks from stalling at PARP1-DNA complexes and TOP1ccs (23–25). The presence of heterozygous missense variants of *FAM111A* that affect only a few amino acids within the trypsin-like peptidase domain of the protein has suggested a gain-of-function (GoF) mechanism as the basis for KCS2.

In the present study, we have identified 2 *FAM111A* missense variants that replace the same critical amino acid, p.Y414C and p.Y414N, that cause KCS2 and OCS, respectively, only when homozygous. Comprehensive analyses of the Y414C recombinant FAM111A protein in mammalian cell lines demonstrate that this protein is more active than the FAM111A^WT^ but is less active than mutant proteins that cause autosomal dominant KCS2. Our study demonstrates that Y414 is a critical residue that affects protein function. Moreover, our work demonstrates that quantitative hypermorphic alleles that encode FAM111A proteins result in variable GoF effects that account for autosomal recessive and dominant forms of KCS2 and OCS.

## Results

### Case reports.

**Figure 1:**
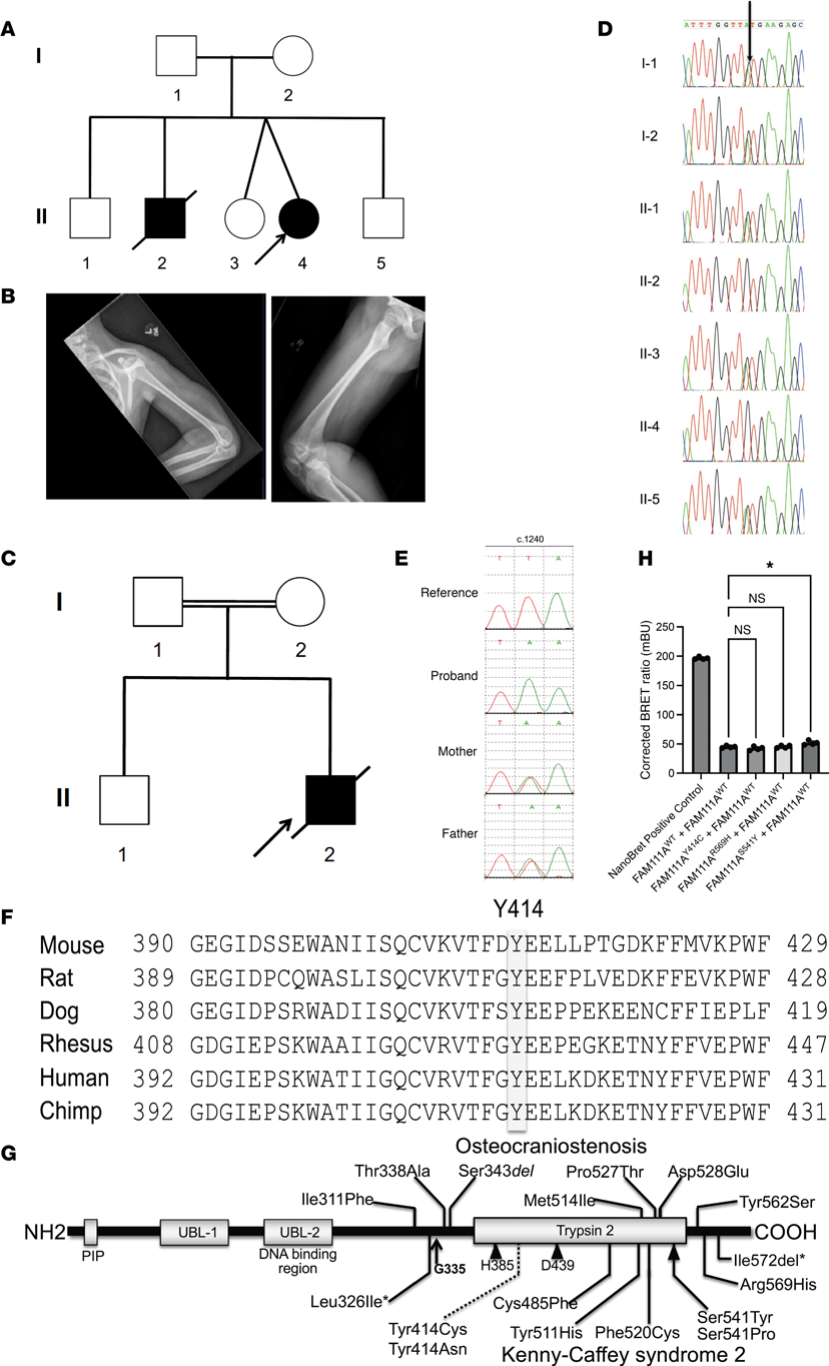
Pedigrees, clinical images, and molecular analysis. (**A**) The pedigree of Family A. (**B**) Radiographic studies of the proband of Family A showing cortical thickening and medullary stenosis in her arm and femur at age 14 years. (**C**) The pedigree of Family B. (**D**). Sanger sequence analysis of *FAM111A* confirmed the c.1241A>G transition; affected patients, II-4 and her brother II-2, are homozygous. Other family members are heterozygous. (**E**) Sanger sequence analysis of *FAM111A* confirmed the c.1240T>A transversion; affected patient II-2 is homozygous. (**F**) The homology comparison of the altered amino acid. The shaded box indicates that FAM111A Y414 residue is conserved among various species. (**G**) Protein domain architecture and pathogenic variants in FAM111A. Residues of the catalytic triad (His385, Asp439, and Ser541) are indicated by arrowheads below the domain (note that Ser541 is also a site of pathogenic variation); a black arrow shows the site of autocatalytic cleavage between residues Phe334 and Gly335; variants associated with KCS2 are shown below the predicted protein while those reported in the more severe GCLEB (also known as OCS) are above. All variants were reported as dominant heterozygotes, except for Leu326Ile and Ile572del (*), which were observed as compound heterozygotes in a case of severe KCS2 or OCS (26). The homozygous Y414N/C variants observed in the current cases is indicated in a dotted line. (**H**) NanoBRET interaction studies. The positive control consisted of a NanoLuc-HaloTag fusion protein that tethers the NanoLuc donor and HaloTag acceptor proteins to ensure efficient energy transfer. One-way ANOVA with Dunnett’s test was performed for statistical analysis. Statistical significance was defined as *P < 0.01. Data are shown as BRET ratio in milliBRET units (mBU). Values represent mean ± SEM.

The proband of Family A (II-4 in Figure 1A) was the product of an uncomplicated twin pregnancy. She was born at 36 weeks with a birth weight of 3.09 kgs and birth length of 45 cm. She had a normal APGAR score (between 7 and 10) at birth, but at 6 hours of age developed respiratory distress. Her initial serum calcium was low (total serum calcium of 7.1 mg/dL and ionized calcium of 1.1 mmol/L). Subsequent evaluation revealed undetectable serum levels of parathyroid hormone (PTH), elevated serum levels of phosphorus, and normal serum levels of magnesium. She was diagnosed with hypoparathyroidism and treated with calcitriol and calcium carbonate. The parents were of Swiss background and denied consanguinity. Her dizygotic female twin was unaffected. There had been a previous fetal loss, and an older male sibling (II-2) had features of KCS and had died at 14 months of age from Respiratory Syncytial Virus infection (RSV). The proband underwent numerous diagnostic evaluations including negative FISH for chromosome 22q11.2 deletion and negative Sanger sequencing of the *TBCE* gene. Nevertheless, a clinical diagnosis of KCS was made based on hypoparathyroidism and other clinical features that included hyperconvex nails, posteriorly rotated ears, a small nose, a large anterior fontanelle, and skeletal defects including mild hypoplasia of the distal phalanges and medullary stenosis of long bones (Figure 1B). She had postnatal growth retardation with extremely short stature (height *z* = –3.2). In addition, she developed left lower extremity lymphedema, bilateral hearing loss, mild neurocognitive defects, tortuous retinal vessels, neutropenia, and recurrent infections. In addition to hypoparathyroidism, other endocrine defects included growth hormone deficiency and transient central hypothyroidism. She underwent menarche at age 14 years and has regular menses. There was no marked improvement in height velocity while taking recombinant growth hormone from age 6 years, and at age 15 years, she reached her adult height of 137 cm (*z* = –4).

Her infections have been notable for frequent sinopulmonary infections requiring 4–5 courses of antibiotics per year. She was hospitalized at age 9 years for mycoplasma pneumonia and sepsis, which required extracorporeal membrane oxygenation (ECMO) and high-frequency ventilation and which was further complicated by cardiac tamponade, pneumothoraxes, and additional infections. She underwent tracheostomy and was discharged after an 8-month hospitalization. At 12 years of age, she had a brief hospitalization for a pulmonary infection with RSV. Since that time, she has remained healthy at home. She has bronchiectasis due to recurrent infections. She was noted to have episodic eosinophilia of 20% and a persistent neutropenia in the range of 600–800 cells/mm^3^. Her immunologic studies are remarkable for intermittently low lymphocyte numbers, low-normal immunoglobulin levels with preserved vaccine responses, and defective T cell proliferative responses (Table 1).

An older brother (II-2 in Figure 1A) died at 14 months and had a history of hydrops fetalis at birth; he had dysmorphic features and respiratory distress due to pleural effusions. His peripheral blood cells were remarkable for immature myeloid forms and progressive thrombocytopenia. He had elevated triglycerides, cholestatic jaundice, and mildly elevated ammonia. Her parents and unaffected sibs (II-1, II-3, and II-5) have normal stature and normal serum levels of calcium and PTH.

The proband in Family B (II-2 in Figure 1C) was the second child born to consanguineous Pakistani parents. The first child is healthy, and there is no history of congenital anomalies in the wider family. Short femurs were identified on an antenatal ultrasound scan from 22 weeks gestation onward. Growth deaccelerated further by 30 weeks gestation, and pleural effusions developed along with more generalized hydrops. The male infant was delivered at 31 weeks due to fetal distress and underwent full resuscitation. The infant died at 11 hours of age despite full active management. Postmortem examination was refused, but photos of the infant identified a small mouth and nose. Chest radiographs done after birth showed thin ribs, a narrow upper chest, and platyspondyly.

### Identification of variants in the FAM111A gene.

We conducted exome sequencing on all living individuals in Family A (Figure 1A); on average, the mean depth of coverage of the exome for each sample was 94×. Two genes (*LAMA3* [MIM 600805] and *SPTBN5* [MIM 605916]) had compound heterozygous variants, and 1 gene (*FAM111A*) had a homozygous variant. Sanger sequencing of DNA from 7 family members, including the deceased child II-2, showed that only the *FAM111A* variant, c.1241A>G, p.Y414C, was homozygous in both the proband and her affected sibling, and it segregated appropriately with affected status (Figure 1D).

DNA samples from the proband of Family B (Figure 1C) and his parents were subjected to genome sequence analysis and revealed only a single candidate — c.1240T>A, p.Y414N in *FAM111A* — that was homozygous in the deceased proband (Figure 1E). Both unaffected parents were heterozygous for this variant; DNA was not available from the older, unaffected sibling.

Analysis of the FAM111A protein showed that the Tyr414 residue is highly conserved among various species (Figure 1F) and is present in a domain with homology to trypsin-like peptidases, including an untested catalytic triad purportedly composed of H385, D439, and S541 (Figure 1G). Tyr414 is in close proximity to L326I, a missense variant previously reported in a patient with autosomal recessive KCS2 (26) as well as multiple heterozygous disease-associated variants.

### FAM111A variant proteins maintain the ability to dimerize.

Since the WT and the constitutively active FAM111A R569H mutant have been shown to interact intermolecularly (23), we aimed to assess the effect of the Y414C variant on oligomeric behavior in a human cell model. We utilized HEK293T cells, a well-established cell model for interactions assays. For this study, the FAM111A^Y414C^ proteins were used for application of a NanoBRET assay studying protein-to-protein interaction after addition of appropriate tags. We found that the amino-terminal position of the fusion tag yielded the best conditions for interaction (data not shown). To minimize the amount of unbound donor and maximize dynamic range for the NanoBRET signal, we transfected HEK293T cells with varying ratios (1:1, 1:10, 1:100, and 1:1,000) of vectors encoding FAM111A^WT^ fusion proteins with amino-terminal labels. The highest fold change was obtained for the donor/acceptor ratio of 1:10. Interaction assays in HEK293T cells showed that the FAM111A^WT^ fusion protein interacted robustly with the FAM111A^WT^ fusion protein as compared with the empty vector (data not shown). Moreover, the FAM111A^WT^ fusion protein interacted equally well with mutant FAM111A fusion proteins containing the Y414C and R569H substitutions and showed modestly greater (*P* < 0.05) interaction with the S541Y substitution (Figure 1H).

### Y414C exerts a mild GoF effect.

**Figure 2:**
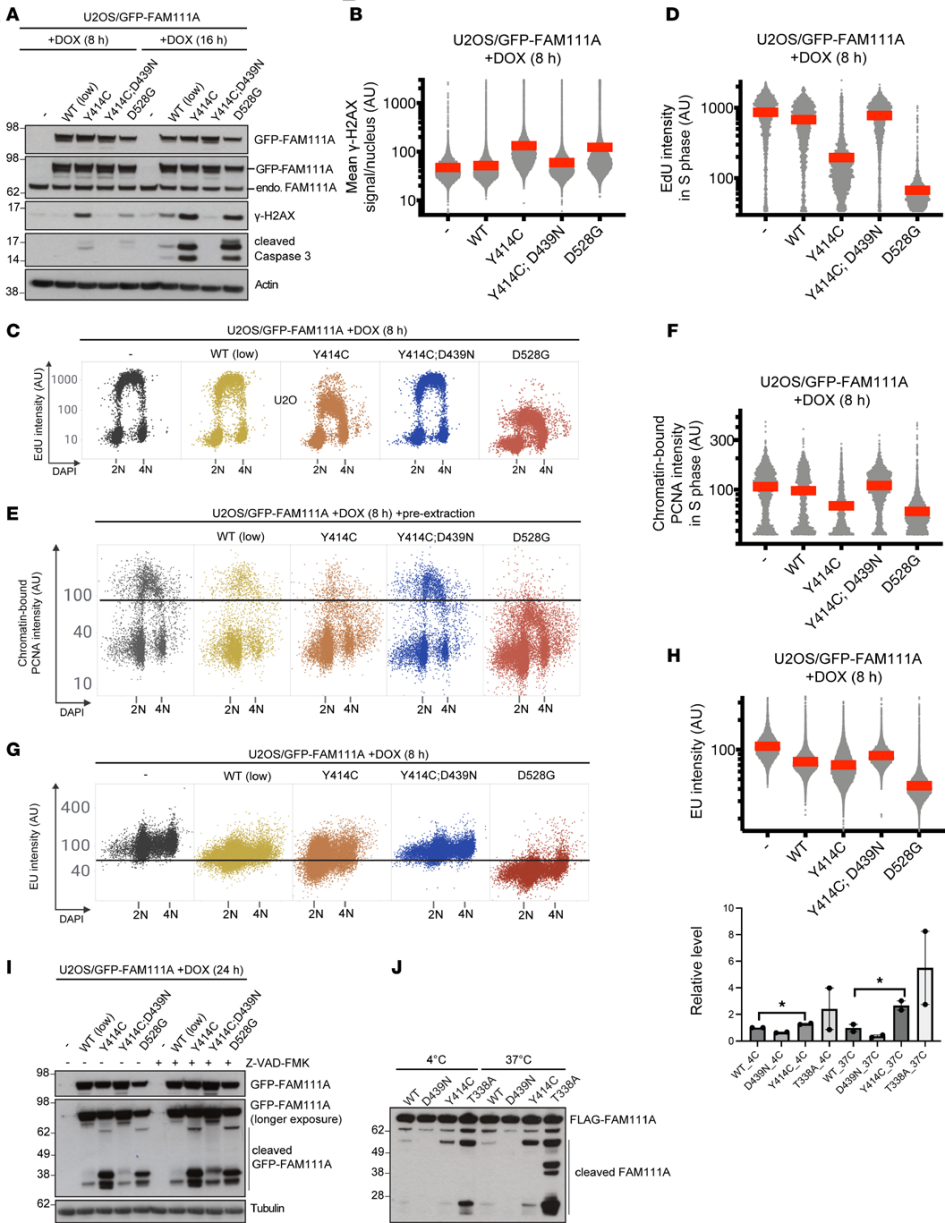
Functional analyses of FAM111A proteins. Data shown are representative of at least 3 independent experiments with similar outcome unless otherwise indicated. (**A**) Immunoblot analysis of U2OS/GFP-FAM111A cell lines left untreated or incubated with doxycycline (DOX) to induce expression of the indicated GFP-FAM111A alleles for 8 or 16 hours. (**B**) U2OS/GFP-FAM111A cell lines treated or not with DOX were fixed, immunostained with γ-H2AX antibody, and subjected to quantitative image-based cytometry (QIBC) analysis of γ-H2AX signal intensity (red bars, mean [AU]; *n* > 10,000 cells per condition). (**C**) U2OS/GFP-FAM111A cell lines treated or not with DOX were pulse-labeled with EdU, stained with DAPI and analyzed for DAPI and EdU signal intensity using QIBC. (**D**) Quantification of data in **C** (red bars, mean; *n* > 2,000 cells per condition). Cells in S phase were identified based on EdU positivity. (**E**) U2OS/GFP-FAM111A cell lines treated or not with DOX were preextracted, fixed, and immunostained with PCNA antibody and were subsequently analyzed by QIBC (*n* > 1,000 cells per condition). (**F**) Quantification of data in **E** (red bars, mean; *n* > 1,000 S phase cells per condition). (**G**) U2OS/GFP-FAM111A cell lines treated or not with DOX were pulse labeled with EU, stained with DAPI, and analyzed by QIBC (red bars, mean; *n* > 9,000 cells per condition). (**H**) Quantification of data in **G** (red bars, mean; *n* > 9,000 cells per condition). (**I**) Immunoblot analysis of U2OS/GFP-FAM111A cell lines treated or not with DOX and the pan-Caspase inhibitor Z-VAD-FMK as indicated. (**J**) Purified recombinant FLAG-FAM111A proteins were incubated at the indicated temperatures for 24 hours, and FLAG-FAM111A autoproteolytic activity was analyzed by immunoblotting with FLAG antibody. *n* = 2 independent experiments. Data indicate the mean ± SEM. *P < 0.05 by 1-tailed *t* test.

Dominant missense variants in human *FAM111A* have been shown to exert their adverse effect on cell and organismal fitness by hyperactivating intrinsic FAM111A protease activity (23, 27). To address whether the recessive Y414C variant in *FAM111A* causes KCS2 by a similar GoF effect, we generated a panel of human U2OS osteosarcoma cell lines conditionally expressing different GFP-tagged patient-associated FAM111A alleles or FAM111A^WT^ at comparable, near-endogenous levels (Figure 2A). We found that expression of both the recessive Y414C and dominant D528G mutants enhanced the effect of GFP-FAM111A on the kinetics and magnitude of apoptosis onset, as evidenced by the apoptotic markers γ-H2AX and cleaved Caspase 3 (Figure 2, A and B). Likewise, FAM111A^Y414C^ expression impaired DNA replication, PCNA chromatin loading, and transcription to a greater extent than FAM111A^WT^, albeit not as potently as the dominant D528G mutant (Figure 2, C–H). Importantly, introducing a catalytically inactivating D439N variant into the FAM111A serine protease domain (SPD) abrogated the effect of the Y414C mutant on DNA replication, transcription, and apoptosis induction (Figure 2, A–H), suggesting that the Y414C variant exerts a GoF effect by amplifying FAM111A proteolytic activity. Supporting this notion, FAM111A cleavage products that were generated via intrinsic protease activity but were insensitive to Caspase inhibition by Z-VAD-FMK were observed for both the D528G and Y414C mutants but not for FAM111A^WT^ (Figure 2I). Consistently, using purified recombinant FAM111A proteins, we observed elevated autoproteolytic cleavage of the Y414C mutant relative to FAM111A^WT^ (Figure 2J). However, the in vitro autoproteolytic activity of FAM111A^Y414C^ was lower than that of a dominant T338A mutant (Figure 2J). Together, these findings show that the Y414C variant possesses increased FAM111A protease activity. Overall, however, the GoF effects are milder than those observed for dominant FAM111A variants, providing a possible explanation for the recessive nature of the Y414C allele.

### In silico modeling of FAM111A protein.

**Figure 3:**
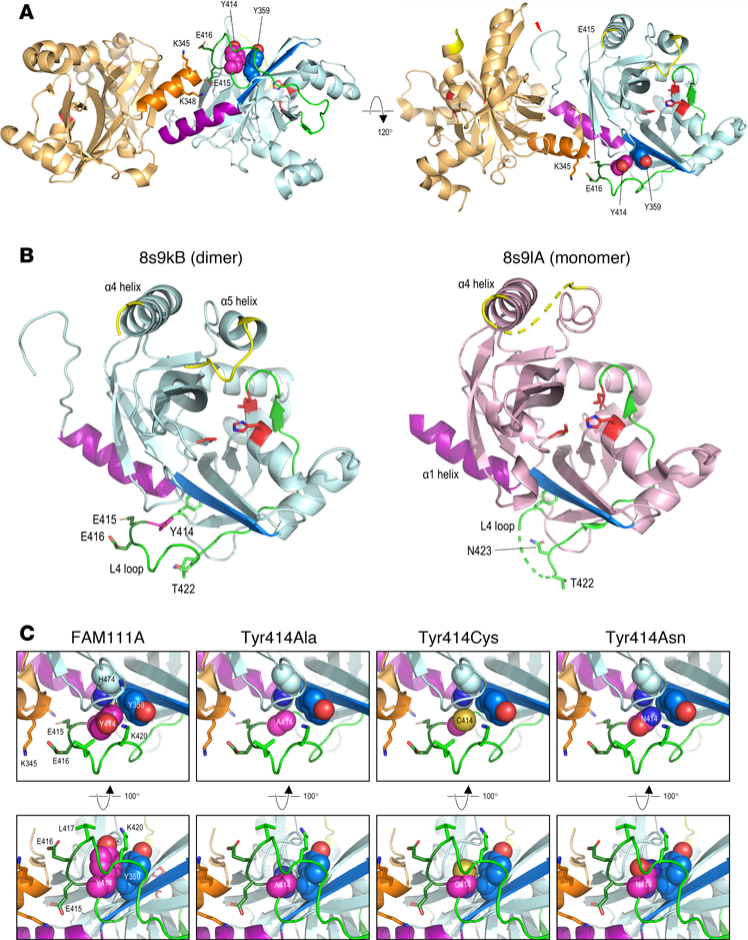
Structure of the FAM111A Trypsin-like peptidase domain and predicted effect of variants at Y414. (**A**) Dimeric structure of the FAM111A Trypsin-like peptidase 853 domain (PDB 8s9k). Monomers are colored by chain (light orange, pale cyan); in both monomers, residues of the α1 dimerization helix (residues 344–355) are show in a darker shade, while residues of the active site (H385, D439, and S541) are colored red. In the right monomer, residues 412–438 of the L4 loop are colored green except for Tyr414, magenta, those of the β1 strand (residues 357–363) blue, and residues of the L8 loop (residues 506–517) yellow; the red flash in the right panel shows the site of autocatalytic cleavage. Sidechains are shown in either stick format or as space-filling spheres as labeled. (**B**) Comparison of the Trypsin-like domain structure in the dimer (8s9k chain B, left) and monomer (8s9l chain A, right); in both parts, residues of the α1 helix (residues 344–356) are colored purple, and those of the L8 loop, between helices α4 and α5, are yellow; residues of the L4 loop are fully resolved in 8s9kB and colored green except for Tyr414, magenta, whereas residues 413–421 are not resolved in 8s9lA due to high mobility. (**C**) Structures are shown as in **A** but expanded to show detail around residue 414 in WT FAM111A or variants as indicated; in the WT structure, the broken red line shows a hydrogen bond between the hydroxyl group of the Tyr414 sidechain and backbone carbonyl oxygen atom of Gly477, shown in stick format; this bond was absent in predicted structures of the variants in which backbone atoms of Gly477 have been omitted for clarity.

To investigate a possible structural basis for functional differences between the recessive Tyr414 variants that we identified and previously described dominant FAM111A variants, we took advantage of the recently reported structure of the FAM111A SPD (24). X-ray crystallography and mutagenesis studies (24) have shown that FAM111A dimerizes via the α1 helix at the N-terminal of the domain (residues 344–354), and that maximal protease activity occurs through an allosteric mechanism involving dimer-sensing residues in the L4 loop (residues 411–438). Importantly, while dimerization is essential for activation of protease activity, it is not required for autocatalytic cleavage. The overall structure of the SPD in the dimer is nearly identical to that of the monomer, with only 2 regions of substantial difference: (a) the region over the dimer-sensing L4 loop, which, in the dimer, forms a stable structure and interacts with the α1 helix of the second chain through 2 salt bridges mediated by Glu415 and Glu416 (Figure 3A), whereas in the monomeric form this loop is mobile, with residues Tyr414-Glu421 unresolved in the structure (Figure 3B); and (b) the region in the L8 loop spanning residues 506–517, and the following α5 helix, which form part of the sidewall of the substrate binding cleft opposite to the dimer interface. In the dimer, Tyr414 makes close contact with Tyr359 in strand β1 (residues 357–363) of the central sheet underlying the catalytic site, thus forming part of the allosteric network connecting the dimer-sensing residues of the L4 loop with the catalytic site (Figure 3C). Modeling of the experimental mutation Tyr414Ala showed that this substitution creates a cavity, largely abolishing interaction with Tyr359, and consistent with this, Palani et al. (24) reported that this mutation had a modest effect on dimerization but severely impaired dimer-dependent activation of protease activity. Notably, Tyr414 is the site of both missense variants observed in our patients; modeling of the Y414C variant suggested partial restoration of contact with Tyr359 compared with the Y414A mutant, while we observed no effect of the Y414C variant on interaction with FAM111A^WT^ protein (Figure 1H). Contact with Tyr359 was further restored in the Y414N variant, though not to the extent seen for Tyr414 in the WT structure.

**Figure 4:**
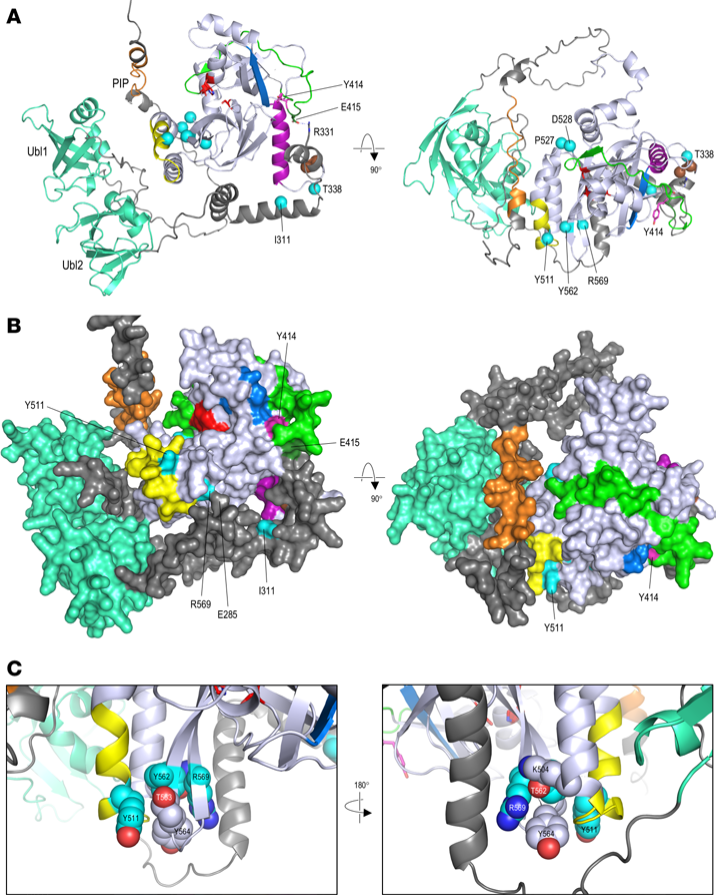
OmegaFold-predicted structure of full-length FAM111A. (**A**) OmegaFold model for full-length FAM111A shown in ribbon format; regions of the Trypsin-like peptidase domain, or SPD, are colored as previously; default coloring of the N-terminal region is dark gray, with the PCNA-binding PIP box colored orange and ubiquitin-like regions 1 and 2 turquoise; sidechains are shown in stick format for Glu415 and Arg331, with the broken red line showing a predicted hydrogen bond. Sidechains are also shown for Tyr414 (magenta) and residues of the catalytic triad (red). Sites of pathogenic KCS/OCS variants are shown as cyan spheres, while Phe334 and Gly335, which flank the site of autocatalytic cleavage, are shown as brown spheres. (**B**) Structures are shown as in **A** but showing the predicted protein surface. (**C**) Structures are shown as in **A** but showing details around residues Tyr511, Tyr562, and Arg569 which lie in close proximity in the 3D structure.

While this modeling shows how variants at Tyr414 might affect allosteric activation of the SPD within the context of the dimeric structure (Protein Data Bank [PDB] 8s9k), it does not directly address how these and other GoF variants result in enhanced autocatalysis of full-length FAM111A. Removal of the N-terminal region has been shown to result in a strong increase in protease activity measured in vitro (24), indicating that that the N-terminal acts directly to mediate autoinhibition of the SPD. However, the absence of structural data for the N-terminal region has prevented full understanding of this phenomenon. We therefore carried out in silico prediction of the structure of full-length FAM111A using OmegaFold (28). Unlike AlphaFold and similar methods that use multiple sequence alignments to construct a consensus model from proteins of homologous sequence, OmegaFold uses a protein language model to predict structure without sequence alignments and, therefore, may be more useful for structural prediction of proteins with limited sequence homology to those in existing databases. The OmegaFold model (Figure 4A) aligned closely to structures of both the monomeric and dimeric forms of the SPD (Root Mean Square Deviation [RMSD] < 0.7Å), indicating the accuracy of the method for modeling of this region. Interestingly, the model also showed the N-terminal to wrap around the SPD, making direct contact at multiple sites (Figure 4B) and suggesting a number of ways by which the N-terminal region might inhibit protease and autocatalytic activity. Firstly, N-terminal regions might act by a simple steric mechanism to restrict substrate access to the catalytic cleft; secondly, binding of the N-terminal on either side of the SPD may serve to hold the catalytic cleft in a more open, inactive state; and thirdly, contacts between the N-terminal region and SPD may have an allosteric effect on protease activity, in a similar but opposing manner to dimerization-dependent activation mediated through the dimer-sensing L4 loop. Notably, the model predicts hydrogen bonding between Glu415, one of the dimer-sensing residues in the L4 loop, and Arg331 in the helix preceding the cleavage site (Figure 4A), suggesting a possible structural basis for variants at Tyr414 to promote autocatalysis. The model also provides a possible explanation for the GoF activity of known pathogenic variants. For example, residues Pro527 and Asp528 lie adjacent in the L9 loop, which forms a flexible hinge between the α5 helix and β8 strand (Figure 4A). Both the Pro527Thr and Asp528Gly variants would be expected to result in increased flexibility of the polypeptide backbone — one through replacement of the constraining proline group, the other through introduction of the sidechain-lacking glycine — and might therefore lead to increased mobility of the α4 and α5 helices relative to the core domain. Since these helices are predicted to lie close to regions of the N-terminal in the model, increased mobility could, thus, act to weaken or disrupt this interaction, leading to a loss of autoinhibition and enhanced autocatalysis. Similarly, residues Tyr511, Tyr562, and Arg569 are predicted to form part of an interaction network between residues of the L8 loop (predicted to form an N-terminal extension to the α5 helix in the OmegaFold model) and strands β10 and β11 of the central β-sheet (Figure 4, A and C), where substitutions might also disrupt the local structure and interaction with the N-terminal; indeed, in the OmegaFold model, there is close contact between Arg569 and Glu285 (Figure 4B), with prediction of hydrogen bonding between the 2 sidechains; this prediction is lost in the Arg569His variant (not shown). Structural analysis in silico predicted the Tyr562Ser variant to be far more destabilizing than the other 2 variants , Tyr511His and Tyr569His, in this region. Alternatively, as the L8 loop undergoes substantial structural rearrangement between the monomeric and dimeric forms of the SPD (Figure 3B), substitutions in these regions might also have an allosteric effect on function, resulting in a higher basal level of catalytic activity and, thus, increased autocatalysis. The hypothesis that pathogenic variants can alleviate N-terminal–mediated autoinhibition also suggests a potential mechanism for variants outside the SPD, specifically Ile311Phe and Thr338Ala, both of which have been reported as pathogenic in OCS and which lie close to the interface between the N-terminal and SPD in the OmegaFold model. Finally, the model suggests that the site of autocatalytic cleavage following Phe334 may be relatively inaccessible for proteolysis and might require interaction of the N-terminal region with its biological targets to bring about its displacement from the SPD and unmasking of the cleavage site, allowing subsequent autocatalytic cleavage and full activation of protease activity only at the intended sites of function.

## Discussion

Heterozygous *FAM111A* variants were originally reported as the cause of KCS2 by Unger et al. (16). These variants, and ones later described in other patients with KCS2 and OCS by other groups (16–20), are mostly de novo missense variants and are clustered in 2 specific regions of the FAM111A protein (29). One cluster lies within the trypsin 2 enzyme domain of the mature FAM111A protein that includes the conserved catalytic triad of histidine 385, aspartate 439, and serine 541 (30), while the second is between the UBLs (ubiquitin-like domains) and the Trypsin 2 domain (Figure 1G). The discrete localization of the heterozygous variants in patients with KCS2 had suggested that these variant FAM111A proteins might represent a GoF effect, and recent experimental studies have supported this hypothesis through the demonstration that these variants possess increased autocleavage activity (23, 27), although the precise targets and mechanisms to explain the diverse phenotypical features of KSC2/OCS remain unknown. An alternative mechanism for the pathobiology of heterozygous *FAM111A* variants in patients with KCS2/OCS was that hyperactive autocleavage activity would cause FAM111A depletion through the formation of FAM111A dimers that can degrade FAM111A^WT^ proteins in trans (23). This model also attempts to explain the molecular mechanism of FAM111A variants in which the active site serine is mutated to proline or tyrosine, such that inactive enzyme might trap and sequester substrates from the FAM111A^WT^ protein, interfering with its function (29). Moreover, while this work was in progress, Bonde et al. (31) reported 2 siblings born to consanguineous parents who manifest features of KCS and who were homozygous for a synonymous *FAM111A* variant, c.81G>A, p.Pro27= that was proposed to affect pre-mRNA splicing that results in skipping of exon 3 in isoform NM_022074.4. Cultured fibroblasts from the affected children showed reduced levels of FAM111A^WT^ transcripts and levels of intact FAM111A protein were similarly reduced, raising the possibility that depletion of FAM111A protein was the mechanism for KCS2 in these patients. Nevertheless, small amounts of variant FAM111A protein were expressed that could have been hyperactive. Moreover, immunoblot analyses of cellular proteins were performed using a monoclonal antibody that was generated using a proprietary immunogen; therefore, no information was provided by the manufacturer about the FAM111A protein epitope that is recognized by the antibody. Thus, it is conceivable that a hyperactive FAM111A protein is expressed that lacks amino acid sequences derived from exon 3 that constrain serine protease activity (31). By contrast, loss of FAM111A function as the basis for the KCS2 phenotype is not supported by the benign appearance of *Fam111a*-KO mice, which are viable without an overt phenotype (32), as well as observations that FAM111A KO is well tolerated at the cellular level (23, 27). Here we present further evidence in support of hyperfunction as the principal basis for KCS2 and describe unique recessive GoF variants.

Our studies raise several important questions. First, how do the recessive variants that we have identified differ functionally from the dominant variants that have been previously described? It is of interest that Y414, the codon altered in affected patients from the 2 kindreds we report here, is located within the dimerization sensor loop L4 of FAM111A, within the SPD, and between the 2 clusters of amino acid replacements that are present in other affected patients. Importantly, disease-associated variants have not previously been reported in this region. Y414 is adjacent to residues E415 and E416, which interact with the α1 chain of the dimeric partner and form a connection between the dimer interface and the active site region through contact with Y359. The interaction between the L4 loop in 1 chain and α1 helix of the other appears to play only a minor role in dimerization itself but is crucial for activation of serine protease activity.

Consistent with the critical role of this codon, an experimental variant, Y414A, has been previously studied and shown to modestly reduce interaction between A414 and Y359 (24), suggesting that, in accordance with the hypothesis that the L4 loop acts as a sensor of dimerization, replacement of Y414 disrupts the allosteric network that regulates dimerization. Although the structure of FAM111A has now been solved in both monomeric and dimeric states, it remains unclear how variants in the L4 loop affect the autocleavage activity, although comparison of the monomer and dimer structures suggests that dimer-induced movement of the L8 loop at the distal end of the substrate binding cleft may be involved in the switch from autocatalytic cleavage to serine protease activity against exogenous substrates. In this context, we hypothesize that disruption of the allosteric network between the dimer-sensing properties of the L4 loop and the activity and specificity of the catalytic site not only impairs dimerization-dependent serine protease activity but allows retention of autocatalytic activity even in the dimeric state. Furthermore, this network appears to be mediated in part by the interaction between Y414 in the L4 loop and Y359 in the central β-sheet, implying a genotype-phenotype relationship between the effect of Y414 variants on the interaction with Y359 at the atomic level and the severity of disease.

Furthermore, our structural analyses predicted that Y562S would be the most destabilizing of the variants in this region, which may explain the association of this variant with a severe skeletal phenotype and OCS (20), whereas Y511H and Y569H are both associated with KCS2. These substitutions might also have an allosteric effect on function that results in a higher basal level of catalytic activity and, thus, increased autocatalysis compared with substitutions at Tyr414. These observations are consistent with the features present in the 2 families that we report here, in which pathogenic manifestations are absent when a patient carries a single Tyr414 variant but KCS2 and OCS occur when a patient carries 2 variant alleles. These aspects of FAM111A function will require further structural, biochemical, and genetic analysis to reveal full details of these processes.

There is a consistent association between autocleavage activity and KCS2/OCS variants, with hyperactivation observed for all dominant *FAM111A* variants that have been assessed (i.e., S541A, R569H, D528G, and T338A) in previous studies (23, 24, 27) and the current study (D528G). In this context, here we show that replacement of Y414 results in a less robust increase in autocleavage activity than occurs in dominant variants, suggesting that the molecular pathobiology of the *FAM111A* variants is a GoF and that the net effect in the biological system is quantitative and gene dose dependent. It is not clear how hyperactivation of FAM111A could result in increased function of the variant protein, but 1 speculation is that increased autocleavage, which occurs between Phe334 and Gly335 (23), could eliminate the N-terminal autoinhibitory region and lead to increased protease activity (Figure 1G). Nevertheless, experimental studies of protease activity provide a less straightforward narrative. For example, dimerization is apparently unaffected by the S541A, R569H, or D528G variants and is modestly affected by Y414A, while protease activity is completely abrogated by the S541A substitution and slightly increased (R569H) or decreased (D528G and Y414A) by other amino acid replacements (24). The relevance of these studies is as yet uncertain, however, since the reported peptidase assays have been performed using an artificial protease substrate (Suc-AAPF-AMC) in vitro, which may not necessarily reflect activity toward a physiological FAM111A substrates in vivo. It will be critical to identify the physiological substrate(s) of FAM111A in order to understand the precise cellular function of FAM111A and, thus, how patient-associated variants undermine fitness.

A second question relates to FAM111A’s physiological function and the effects of increased protein activity. FAM111A has been shown to act as a host range restriction factor through its specific interaction with the C-terminal region of the SV40 large T (LT) antigen (30), and FAM111A can restrict SV40 and adenovirus replication (30, 33). FAM111A has additional chromatin-associated functions that are perhaps more relevant to the development of the broad spectrum of abnormalities in patients with KCS2/OCS. FAM111A is recruited to sites of nascent DNA replication and transcription via its PCNA-interacting peptide (PIP) box (Figure 1G) and promotes S phase entry and DNA synthesis (34). FAM111A has been shown to either block (27, 33, 34) or enhance (34, 35) DNA replication. Consistent with this, FAM111A has recently been shown to promote fork progression through chemically induced DNA-binding protein crosslinks (23). Here we showed that FAM111A^Y414C^ expression impaired DNA replication, PCNA chromatin loading, and transcription to a greater extent than FAM111A^WT^, albeit not as potently as the dominant D528G mutant (Figure 2, C–H). Regrettably, due to laboratory closures, we were unable to generate similar functional data for the Y414N variant, which represents a potential limitation to our experimental evidence. Importantly, introducing a catalytically inactivating D439N variant into the FAM111A SPD abrogated the effect of the Y414C mutant on DNA replication, transcription, and apoptosis induction (Figure 2, A–H), suggesting that the Y414C variant exerts a GoF effect by amplifying FAM111A proteolytic activity. The relevance of this defect to the development of the broad phenotypical features of KCS2 remains uncertain, however.

A third question is related to the phenotype of KCS2. It has been proposed that patients with *FAM111A* variants do not show developmental delay (16). Nevertheless, Cavole et al. described a patient with autosomal dominant KCS2 having intellectual disability and microcephaly (36). Although the 2 cases described by Eren et al. (26) with compound heterozygous *FAM111A* variants did not manifest neurological developmental delay, they died at less than 4 months of age from septicemia. By contrast, the proband in Family A had evidence of moderate developmental delay. These results indicate that neurocognitive delay can also be a component of KCS2 due to *FAM111A* variants. In addition to the multiple developmental and endocrinological defects that are associated with KCS2, patients also have an increased susceptibility to infection that has been attributed to a putative defect in T cell immunity (4). *FAM111A* is widely expressed in many cells and tissues including CD4^+^ T cells, CD8^+^T cells, CD19^+^ B cells, NK cells, and monocytes, as demonstrated in previous ENCODE RNA-Seq data (37) and proteomic data (38), supporting a potential role in immunologic function. The proband in Family A had a medical history of recurrent, severe infections that had suggested an immune deficiency, but when she was initially evaluated as an infant, she was found to have normal T cell numbers and normal levels of immunoglobulins. Subsequent evaluations demonstrated intermittent mild neutropenia and T cell lymphopenia (Table 1) that likely reflect a defect in the nature and function of NK cell microtubules (Jordan S. Orange, Department of Pediatrics, Columbia University College of Physicians and Surgeons, New York, New York, USA, personal communication). Overall, these findings indicate that increased FAM111A activity results in functional deficiencies that resemble those present in lymphoblastoid cells from patients with KCS1 with *TBCE* variants (16). Nevertheless, we lack a complete understanding of FAM111A function and its role in the KCS2 phenotype. FAM111A localizes to both the cytoplasm and nucleus, and it is widely expressed in adult and fetal tissues, including bone, parathyroid, spleen, kidney, lung, ovary, liver, pancreas, and fetal liver. Less abundant expression is present in adult heart, skeletal muscle, and testis and in all fetal and adult brain regions (38). Based on the phenotype of KCS2, FAM111A is believed to have a critical role in intracellular pathways regulating skeletal development, linear growth, and parathyroid gland development and regulation. Nevertheless, the specific substrates and/or molecular targets for FAM111A remain unknown. In vitro experiments on FAM111A-KO cell lines indicate a minimal effect on cell viability but an increased sensitivity to specific agents inducing DNA replication stress (23, 27). However, functional evidence for the pathogenicity of FAM111A variants is still lacking, and the molecular mechanisms by which variants also cause variable severity of OCS and KCS have not yet been elucidated.

In conclusion, here we show that *FAM111A* variants that encode proteins with milder degrees of hyperactivation can cause KCS2/OCS only when biallelic, as heterozygous relatives of the 2 probands we describe lack features of KCS2 or OCS. Our work confirms and extends the recent case report of 2 siblings with immune deficiency and KCS2 who were compound heterozygotes for *FAM111A* variants (c.976T>A; p.L326I and in-frame deletion variant c.1714_1716del; p.Ile572del, rs779963813) (26). These 2 infants died of septicemia, and no functional studies were reported (26). Taken together, our findings suggest that genetic variants of *FAM111A* that encode proteins with variable increases of protease activity can provide a mechanistic basis for both dominant and recessive inheritance of KCS2. These findings challenge the present notion that KCS1 and KCS2 can be distinguished by the apparent mode of transmission and provide confirmation for the notion that *FAM111A* variants lead to a GoF as the pathogenic mechanism. The *FAM111A* variants may lead to immune deficiency when severe. The pathophysiology of abnormal FAM111A function as a cause of KCS/OCS remains to be fully elucidated.

## Methods

### Sex as a biological variable.

We studied both male and female individuals, and similar findings are reported for both sexes.

### Patient recruitment.

We isolated genomic DNA from peripheral blood mononuclear cells from the probands and their available relatives, and from hepatic tissue obtained at autopsy of the affected brother of the proband in Family A (II-2 in Figure 1A), using standard techniques. The diagnoses of KCS and OCS were based on clinical and radiographic criteria.

### Identification of variants and bioinformatic analysis.

The exome was captured for the proband (II-4 in Figure 1A) as well as her parents (I-1 and I-2) and 3 unaffected siblings (II-1, II-3, and II-5) in Family A using the Agilent SureSelect Human All Exon V3 kit (Agilent Technologies). Captured DNA was sequenced using a HiSeq 2000 sequencer (Illumina) with 101 bp pair-end reads with 8 nt indices. Image analysis and base calling were performed using HiSeq Control Software/Real-time analysis and CASAVA 1.8.2 (Illumina) with default parameters as we have previously described (39, 40). All the raw reads were aligned to the reference human genome (UCSC hg19) using the Burrows-Wheeler Aligner (BWA) (41), and PCR duplicates were marked and removed with Picard (https://broadinstitute.github.io/picard/). Local realignment of reads in the indel sites and base quality recalibration were performed with the Genome Analysis Tool Kit (GATK, version 2.3) (42). The kinship coefficient was calculated between every 2 samples via KING (43) to confirm reported relationships.

We performed genome sequencing using DNA from the proband and both parents in Family B. We utilized a gene-agnostic bioinformatics pipeline to filter variants based on their mode of inheritance in the proband. We reviewed de novo and recessive variants in the proband. The *FAM111A* gene was placed onto GeneMatcher to find similar patients.

By adjusting minor allele frequency (MAF) to 0.5%, we also examined possible inheritance by a de novo dominant model. Under recessive modes of inheritance (genes carrying rare homozygous variants or 2 rare compound heterozygous variants), we excluded variants that were: (a) synonymous variants; (b) with MAF > 0.01 in either 1000 Genomes Project or 6,503 exomes from the Exome Sequencing Project (ESP6500SI; https://genome.ucsc.edu/cgi-bin/hgTables?db=hg19&hgta_group=varRep& hgta_track=evsEsp6500&hgta_table=evsEsp6500&hgta_doSchema=describe+table+schema); (c) with other occurrences (>5) in the homozygous state in our in-house exomes. Validation of the variant candidates was performed by targeted Sanger sequencing of DNA.

### FAM111A interaction studies.

We used a nanoluciferase-based bioluminescence resonance energy transfer (NanoBRET) assay in HEK293T cells to assess interaction of WT and the FAM111A^Y414C^ fusion proteins (FAM111A^WT^ and FAM111A^Y414C^, respectively). FAM111A^WT^ proteins were tagged with either NanoLuc donor or HaloTag acceptor tags at either the amino or carboxy terminus of the protein by PCR-based subcloning the *FAM111A* cDNA into pFN21A Halo and pFN31K Nluc vectors (Promega Corporation), which provide constitutive protein expression in mammalian cells using the human cytomegalovirus immediate-early enhancer/promoter. The resulting donor/acceptor combinations were analyzed to identify the optimal pair for signal generation according to the manufacturer’s protocol and the NanoBRET Protein:Protein Interaction System (Promega Corporation). We used PCR-based site directed mutagenesis as previously described (44) to generate FAM111A^Y414C^ fusion proteins in which a HaloTag protein tag was present in the amino terminus. Interaction assays were performed using HEK293T cells that were cultured and maintained at 37°C, 5% CO_2_ in complete medium (DMEM containing 0.3 mg/mL glutamine, 100 IU/mL penicillin, and 100 μg/mL streptomycin; Thermo Fisher Scientific) supplemented with 10% FCS. Transient transfections were carried out 24 hours after seeding cells in 12 well-plates using the FuGENE 6 (Promega) transfection reagent according to the manufacturer’s instructions. Cells were harvested with 0.05% Trypsin-EDTA 24 hours after transfection and seeded into poly-L-lysine–coated (Sigma-Aldrich) white 96-well plates (Costar 3917) at 80,000 cells/well in phenol red-free DMEM containing 25 mM HEPES, 0.3 mg/mL glutamine, 100 IU/mL penicillin, and 100 μg/mL streptomycin supplemented with 5% FCS. After 24 hours, the NanoLuc substrate furimazine (Promega) was added to a final concentration of 10 μM ,and light emission was measured at 37°C using the filters 450 nm (80 nm bandpass) and 610 nm (longpass) on a multilabel plate reader. Raw BRET ratios were calculated by dividing the 610 nm emission (acceptor) by the 460 nm emission (donor).

### FAM111A protein biochemical studies.

pcDNA4/TO/eGFP and pYES-FLAG-SNAP-TopII plasmids expressing either FAM111A^WT^ or FAM111A^Y414C^ were generated and used to create human U2OS cell lines stably expressing GFP-tagged FAM111A^WT^ and FAM111A^Y414C^ as previously described (27). Positive clones were selected by incubation in cell medium containing 5 μg/mL blasticidin S (Invitrogen) and 400 μg/mL Zeocin (Invitrogen) for 14 days. Unless otherwise indicated, the following drug concentrations were used: Z-VAD-FMK (50 μM, ab120487, Abcam) and doxycycline (1 μg/mL, Sigma-Aldrich).

Immunoblotting was performed as previously described (45) using the indicated antibodies (see below). For in vitro autocleavage assays, recombinant proteins were incubated in EBC buffer (50 mM Tris [pH 7.5], 150 mM NaCl, 1 mM EDTA, 0.5% NP40, 1 mM DTT; MilliporeSigma).

For immunofluorescence and high-content imaging analysis, cells were preextracted in PBS containing 0.2% Triton X-100 for 2 minutes on ice, before fixation with 4% formaldehyde for 15 minutes. If cells were not preextracted, they were subjected to a permeabilization step with PBS containing 0.2% Triton X-100 for 5 minutes and incubated with primary antibodies diluted in 1% BSA-PBS for 1 hour at room temperature. Following staining with secondary antibodies (Alexa Fluor; Life Technologies) and DAPI (0.5 μg/mL, DNA staining) diluted in 1% BSA-PBS for 1 hour at room temperature, cells were mounted onto glass slides using ProLong Gold Antifade (Invitrogen).

For EdU staining, cells were treated with EdU (10 μM) for 30 minutes before fixation and then stained using the Click-iT Plus EdU Alexa Fluor 647 Imaging Kit (Invitrogen) according to the manufacturer’s instructions before incubation with primary antibodies. Fluorescent staining of newly transcribed RNA was performed via metabolic labeling with 5-ethynyluridine (EU); cells were treated with EU (1 mM) for 60 minutes before fixation and then stained using the Click-iT RNA Alexa Fluor 594 Imaging Kit (Invitrogen) according to the manufacturer’s instructions.

Quantitative image-based cytometry (QIBC) was performed as described (46). Briefly, cells were fixed, permeabilized, and stained as described above. Images were acquired with a scanR inverted high-content screening microscope (Olympus) equipped with wide-field optics, UPLSAPO dry objectives (×20, 0.75 NA), fast excitation and emission filter-wheel devices for DAPI, FITC (fluorescein isothiocyanate), Cy3 and Cy5 wavelengths, an MT20 illumination system, and a digital monochrome Hamamatsu ORCA-R2 CCD camera. Automated and unbiased image analysis was carried out with the ScanR analysis software (version 2.7.1). Data were exported and processed using Spotfire software (version 10.5.0; Tibco).

### Purification of recombinant FAM111A proteins.

The purification of FLAG-tagged recombinant FAM111A was described previously (27). Briefly, JEL-1 yeast that had been transformed with FLAG-FAM111A expression plasmid were grown in SC-URA medium (MP Biomedicals) containing 2% glucose for 36 hours at 30°C and, subsequently, in SC-URA medium containing 2% raffinose for 24 hours at 30°C. The culture was then placed in YEP medium containing 2% raffinose and grown until OD 0.8. FLAG-FAM111A protein expression was induced by growth in the presence of 2% galactose for 24 hours at 20°C. Cells were collected and resuspended in lysis buffer containing 50 mM Tris (pH 7.5), 500 mM NaCl, 10% glycerol, 1 mM EDTA, and 0.1 mM DTT (MilliporeSigma). Glass beads were added to the resuspended cells, which were lysed by vortexing and cleared by centrifugation at 25,000*g* at 4°C for 30 minutes. The cleared lysate was incubated with FLAG M2 resin (Sigma-Aldrich) and incubated at 4°C for 2 hours. After extensive washing, FLAG-FAM111A was eluted in lysis buffer supplemented with FLAG peptide (0.5 mg/mL). The elute fractions were run on a 4%–12% NuPAGE Bis-Tris protein gel (Invitrogen) and stained with Instant Blue Coomassie Protein Stain (Expedeon). Fractions were concentrated on Microcon-30 kDa Centrifugal Filters (MilliporeSigma), snap frozen in liquid nitrogen, and stored at −80°C.

### Antibodies.

Antibodies used for immunoblotting included pan-actin (MAB1501, clone C4, MilliporeSigma, 1:20,000), Cleaved Caspase-3 (Asp175, 9661, Cell Signaling Technology, 1:1,000), FAM111A (ab184572, Abcam, 1:1,000), FLAG (A00187, GenScript, 1:1,000), GFP (11814460001, Roche, 1:500; sc-8334, Santa Cruz Biotechnology Inc., 1:1,000), γ-H2AX (2577L, BioNordika, 1:1,000), and α-tubulin (T9026, Sigma-Aldrich, 1:5,000). Antibodies used for immunofluorescence included γ-H2AX (05-636, clone JBW301, MilliporeSigma, 1:500), PCNA (2037, Triolab Immunoconcepts, 1:500).

### In silico modeling of FAM111A protein.

Molecular modeling was carried out using PDBs 8s9k and 8s9l, structures of FAM111A residues 332–600 (Trypsin-like peptidase domain and autocleavage site) in the dimeric and monomeric states, respectively (24). Variants were introduced in silico using FoldX (47). Docking of the peptidase domain (residues 343–587) with an autocleavage substrate peptide (FAM111A residues 330–341) was performed using AlphaFold 3 (48) via the AlphaFold web server (https://alphafoldserver.com/). Reconstruction of residues in loop regions L4 and L8 of the monomeric protease domain structure was performed using the I-TASSER server (https://seq2fun.dcmb.med.umich.edu//I-TASSER/) (49), with PDB 8s9l specified as template. Modeling of full-length FAM111A was performed using OmegaFold (28) via the COSMIC2 web portal (https://cosmic2.sdsc.edu) (50). Structures were visualized in PyMOL (PyMOL Molecular Graphics System, Version 3.0.2, Schrödinger LLC).

### Statistics.

Data analysis and graphing were performed using Prism 5.0 (GraphPad Software). Statistical analysis was largely performed on repeated experiments with matched experimental conditions using 2-way ANOVA with Dunnett’s multiple-comparison tests. Mean data from multiple independent experiments were analyzed using statistical tests based on a normal distribution. Specific information on the number of separate experiments performed and the number of replicates obtained in each individual experiment is provided in each figure legend. The level of significance was considered to be *P* < 0.05.

### Study approval.

All patients provided written informed consent or assent for participation in the study. The study was approved by the IRB of The Children’s Hospital of Philadelphia (IRB 12-008863, Skeletal [Bone] and Mineral Metabolism [BAM] Biorepository). Genetic testing in Family B was conducted in accordance with the requirements for clinical diagnostic testing provided through the National Health Service in England, United Kingdom.

### Data availability.

Supporting data for full values underlying the data presented in the graphs are provided in the Supplemental Supporting Data Values file (supplemental material available online with this article; https://doi.org/10.1172/jci.insight.186862DS1).

## Author contributions

DL and MAL conceptualized the study. DL, RCC, LP, and HH conducted molecular analysis. JE and MAL conducted clinical evaluation of the affected individuals. DL, NM, EE, SH, YD, KES, and MAL performed functional assessment and validation. DL and MAL wrote the original draft of the manuscript. All authors reviewed and edited the manuscript.

## Supplementary Material

Unedited blot and gel images

Supporting data values

## Figures and Tables

**Table 1:**
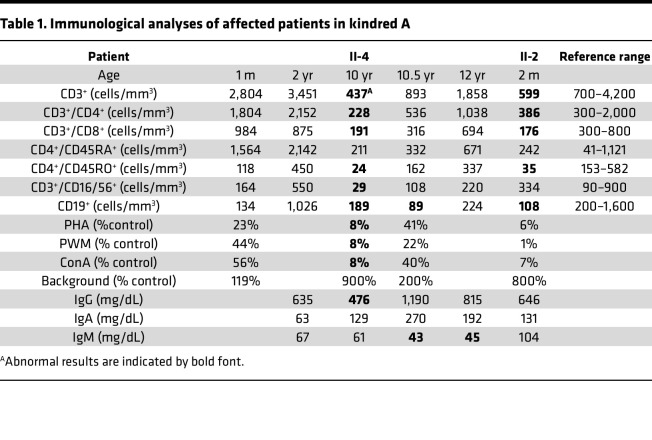
Immunological analyses of affected patients in kindred A

## Version 1 02/11/2025

In-Press Preview

## Version 2 03/24/2025

Electronic publication
