# Supplementary figures and images for: Quantitative hypermorphic FAM111A alleles cause autosomal recessive Kenny-Caffey syndrome type 2 and osteocraniostenosis
Source: JCI Insight. 2025 Feb 11;10(6):e186862. doi: 10.1172/jci.insight.186862 (PMC11949059; doi:10.1172/jci.insight.186862)

Fig 2A:

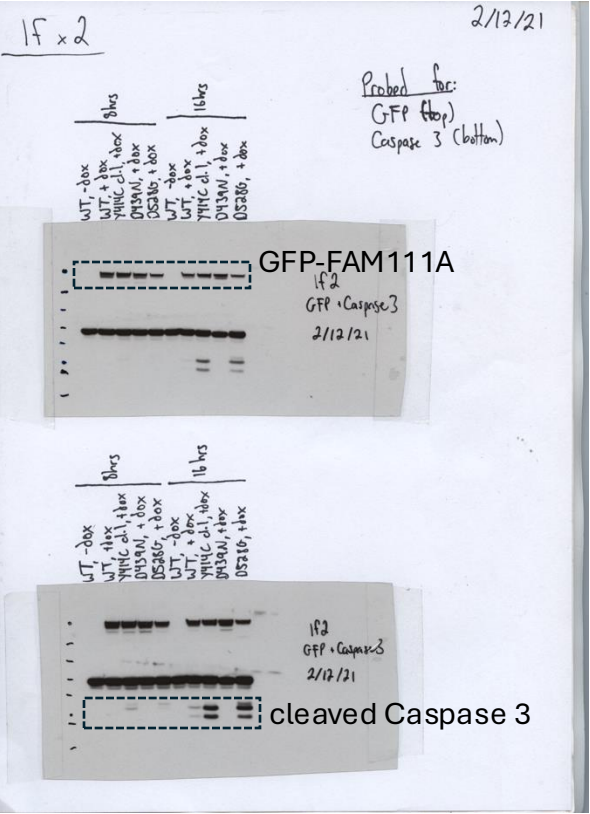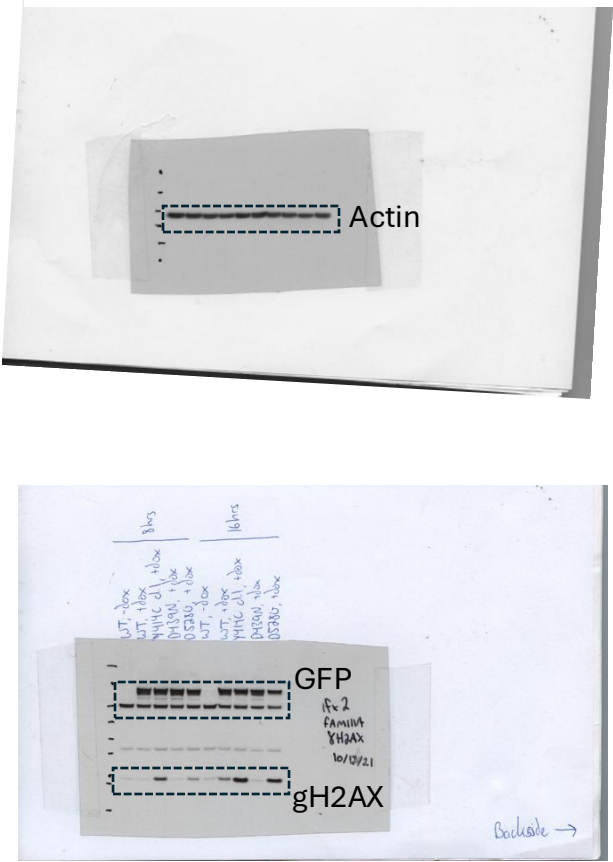

Fig 2l:

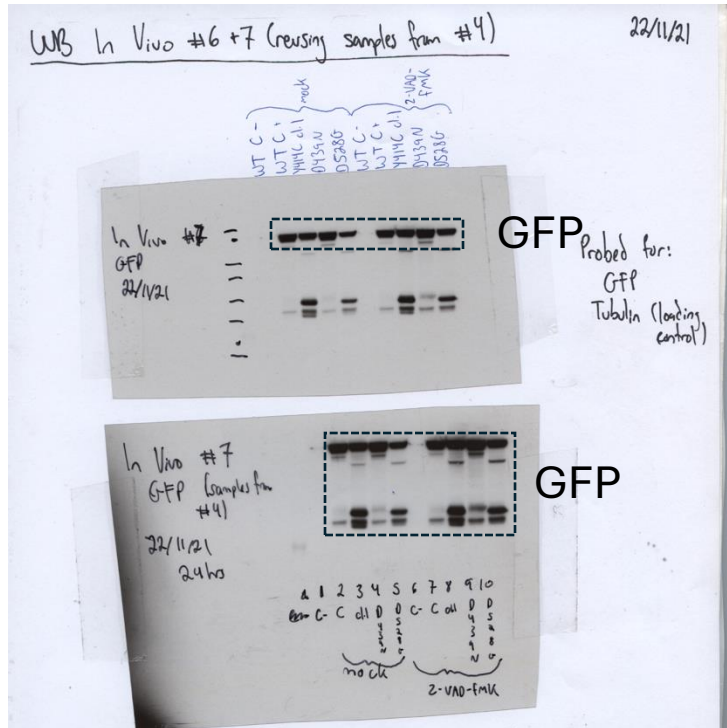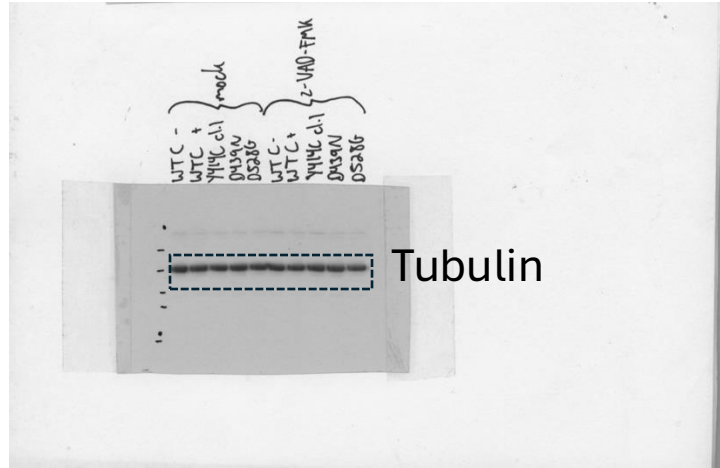

Fig 2J:

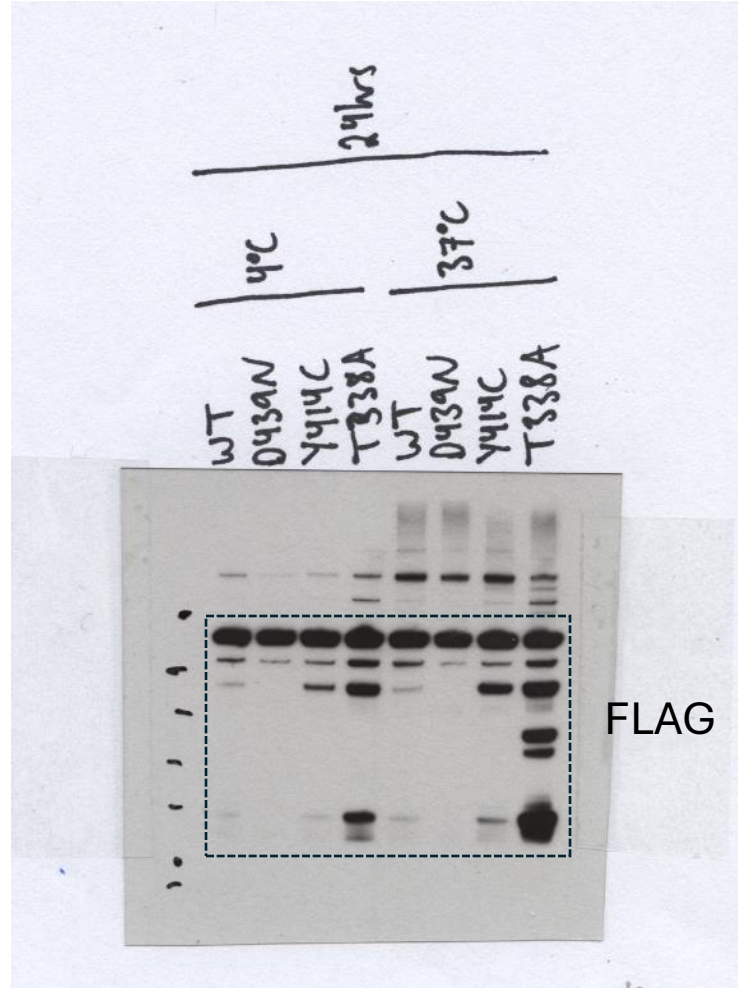

Supplement: Unedited blot and gel images [file jciinsight-10-186862-s219.pdf]
